# Gut Microbiota in Bipolar Depression and Its Relationship to Brain Function: An Advanced Exploration

**DOI:** 10.3389/fpsyt.2019.00784

**Published:** 2019-10-29

**Authors:** Qiaoqiao Lu, Jianbo Lai, Haifeng Lu, Chee Ng, Tingting Huang, Hua Zhang, Kaijing Ding, Zheng Wang, Jiajun Jiang, Jianbo Hu, Jing Lu, Shaojia Lu, Tingting Mou, Dandan Wang, Yanli Du, Caixi Xi, Hailong Lyu, Jingkai Chen, Yi Xu, Zhuhua Liu, Shaohua Hu

**Affiliations:** ^1^Department of Psychiatry, the First Affiliated Hospital, College of Medicine, Zhejiang University, Hangzhou, China; ^2^Department of Psychiatry, Hangzhou Seventh People’s Hospital, Hangzhou, China; ^3^The Key Laboratory of Mental Disorder Management of Zhejiang Province, Hangzhou, China; ^4^Brain Research Institute, Zhejiang University, Hangzhou, China; ^5^State Key Laboratory for Diagnosis and Treatment of Infectious Diseases, First Affiliated Hospital, Zhejiang University School of Medicine, Hangzhou, China; ^6^The Melbourne Clinic, Department of Psychiatry, University of Melbourne, Melbourne, VIC, Australia; ^7^Department of Children and Adolescents’ Psychology, Hangzhou Seventh People’s Hospital, Hangzhou, China; ^8^Center of Mental Health, General Hospital of Ningxia Medical University, Yinchuan, China

**Keywords:** bipolar depression, gut microbiota, brain function, immune function, quetiapine treatment

## Abstract

The mechanism of bipolar disorder is unclear. Growing evidence indicates that gut microbiota plays a pivotal role in mental disorders. This study aimed to find out changes in the gut microbiota in bipolar depression (BD) subjects following treatment with quetiapine and evaluate their correlations with the brain and immune function. Totally 36 subjects with BD and 27 healthy controls (HCs) were recruited. The severity of depression was evaluated with the Montgomery-Asberg depression rating scale (MADRS). At baseline, fecal samples were collected and analyzed by quantitative polymerase chain reaction (qPCR). T lymphocyte subsets were measured to examine immune function. Near-infrared spectroscopy (NIRS) was used to assess brain function. All BD subjects received quetiapine treatment (300 mg/d) for four weeks, following which the fecal microbiota and immune profiles were reexamined. Here, we first put forward the new concept of brain-gut coefficient of balance (B-G_CB_), which referred to the ratio of [oxygenated hemoglobin]/(*Bifidobacteria* to *Enterobacteriaceae* ratio), to analyze the linkage between the gut microbiota and brain function. At baseline, the CD3^+^ T cell proportion was positively correlated with log_10_ Enterobacter spp count, whereas the correlativity between the other bacteria and immune profiles were negative. Log_10_ B-G_CB_ was positively correlated with CD3^+^ T cell proportion. In subjects with BD, counts of *Faecalibacterium prausnitzii*, *Bacteroides–Prevotella group*, *Atopobium Cluster*, *Enterobacter spp*, and *Clostridium Cluster IV* were higher, whereas the log_10_ (B/E) were lower than HCs (B/E refers to *Bifidobacteria* to *Enterobacteriaceae* ratio and represents microbial colonization resistance). After treatment, MADRS scores were reduced, whereas the levels of *Eubacterium rectale*, *Bifidobacteria*, and B/E increased. The composition of the gut microbiota and its relationship to brain function were altered in BD subjects. Quetiapine treatment was effective for depression and influenced the composition of gut microbiota in patients.

**Clinical Trial Registration:** http://www.chictr.org.cn/index.aspx, identifier ChiCTR-COC-17011401, URL: http://www.chictr.org.cn/listbycreater.aspx.

## Introduction

Bipolar disorder caused a high global disease burden (1) with a lifetime prevalence of 1.0% for bipolar-I disorder, 1.1% for bipolar-II disorder, and 2.4% for subthreshold bipolar disorder. The pathogenesis of bipolar disorder is unclear. Its diagnosis is based on clinical symptoms. Hence, it is frequently misdiagnosed as major depressive disorder (2, 3). The misdiagnosis results in poor therapeutic outcomes (2). Therefore, it makes great sense to study the mechanism of bipolar disorder, search for biomarkers, and find novel therapies.

Gut microbiota plays an increasingly important role in the onset and progress of psychiatric disorders. Brain-gut axis is the highway on which biochemical molecules travel between the brain and the gut. These biochemical molecules are involved in immune system, endocrine system, and the vagus nerve (4, 5). Many of these molecules can be produced by gut microbiota directly or indirectly. Therefore, we suspect that gut microbiota triggers bipolar disorder through brain-gut axis is one of the potential mechanisms of bipolar pathogenesis. This includes immune, neural, and endocrine pathways. These pathways work in tandem and is also a complex whole that communicates with each other on the way to bipolar disorder. Gut microbiota is associated with activation of pro- or anti-inflammatory responses in central nervous system (CNS) (6). Short-chain fatty acids (SCFAs) are products of carbohydrates fermented by gut bacteria. SCFAs are protective factors for inflammatory status in both peripheral and CNS (7, 8). Other gut metabolites and microbial components also contribute to inflammatory modulation, such as polyamines, polysaccharide A, formyl peptides, and D-glycero-β-D-mannoheptose-1,7-bisphosphate (8). Some bacteria, such as *Lactobacillus* strain, regulate emotion *via* vagus nerve (9). Enteroendocrine cells release mediators, such as 5-hydroxytryptamine (5-HT), cholecystokinin, glucagon-like peptide 1, peptide YY, and ghrelin. These mediators are regulated by intestinal nutrient status. They stimulate vagal afferent neurons signaling to CNS to regulate food intake behaviors in mood disorder patients (10). In particular, 5-HT plays an important role in the regulation of emotion (11). Hypothalamic-pituitary-adrenal axis is also influenced by gut microbiota (12), communicates with immune system (13), and is involved in emotion regulation (12).

A growing body of research finds that gut microbiota in bipolar patients is different from that in normal persons. In bipolar disorder patients, *Clostridiaceae* is found more abundant than that in HCs (14). Two studies observed the decline of *Faecalibacterium* in bipolar disorder subjects (15, 16), which is related to better self-reported health states (16). The presence of genus *Flavonifractor* is a risk factor for bipolar disorder and is confounded by female sex and smoking (17). As a dangerous factor, genus *Flavonifractor* may induce inflammation and oxidative stress to harm its host (17). In patients with bipolar disorder, negative correlations are found between *Lactobacillus* counts and sleep, and between *Bifidobacterium* counts and serum cortisol levels, indicating these two bacteria impact exact depressive symptoms (18). Special bacteria changes in bipolar patients in different researches are not completely coincident. Compared with HCs, phylum *Actinobacteria* and class *Coriobacteria* are more abundant in bipolar patients, whereas class *Ruminococcaceae* is more abundant in HCs (15). Two studies have analyzed gut microbiota in bipolar patients who received psychotropic medications therapy. One study shows that atypical antipsychotics treated females have lower species richness when compared with nonatypical antipsychotics treated females (19). However, this is not significant in male patients (19). In this study, the atypical antipsychotics include clozapine, olanzapine, risperidone, quetiapine, asenipine, ziprasodone, lurasidone, aripiprazole, paliperidone, and iloperidone (19). The other study identifies 30 microbial markers in bipolar depression patients, which are different from HCs, and finds the alteration of gut microbiota composition following quetiapine monotherapy (20). Moreover, 10 microbial markers are identified from the responders of patients (20).

As yet, no study has explored the connection among the brain function, gut microbiota, and immune function in BD population. This study not only attempts to compare the gut microbiota between BD and HCs but also to find this potential connection. The results will be a supplement to the brain-gut axis in the psychiatric field. Besides that, this study also tries to repeat the effects of quetiapine treatment on gut microbiota in BD patients.

## Materials and Methods

### Study Design and Subjects

This study was approved by the Ethics Committee of the First Affiliated Hospital, Zhejiang University School of Medicine (a quick review of scientific research, No.397, 2017, see Supplement for the original document) and was performed in accordance with the Helsinki Declaration of 1975. The clinical trial registry number was ChiCTR-COC-17011401. All participants were provided informed consents before being recruited into the study (for adolescents, consents were provided by their guardians). The privacy rights of the recruited subjects were taken seriously.

Altogether, 36 subjects with BD were recruited from the inpatient and outpatient departments of the First Affiliated Hospital, Zhejiang University School of Medicine from May to October 2017. Twenty six patients suffered from bipolar II, and 10 patients had bipolar I. The age of the subjects ranged from 14 to 57 years old. All subjects met the following criteria: (a) BD was diagnosed by two psychiatrists according to the Structured Clinical Interview for DSM-IV-TR disorders (SCID); there was no severity requirement for depression; (b) BD subjects who had not received any treatment (17 patients) or medications had been stopped for more than 3 months (19 patients stopped medications because of the recovery from the disease or the intolerance of side effects). The exclusion criteria included (I) comorbidity of gastrointestinal disease, infectious disease, fever, and other physical diseases; (II) suffering from autoimmune disease, endocrine disease, and other mental disorders; (III) a history of using antibiotics, probiotics, prebiotics, synbiotics, or yoghurt within a month; (IV) taking lactulose, prokinetic drugs, or antibiotics treatment in the last month; and (V) any history of substance abuse, including nicotine dependence, caffeine use, and others.

In total, 27 age-, gender-, marriage-, and BMI-matched HCs were recruited from five communities in Hangzhou, China. All HCs were excluded if they met any of the exclusion criteria of the BD group or the following criteria: irregular bowel frequency (regular frequency refers to daily or every other day); history of suicide attempts; Montgomery-Asberg depression rating scale (MADRS) (21) score greater than 8 points (22); young mania rating scale (YMRS) (23) score greater than 5 points; and any positive diagnosis on the mini-international neuropsychiatric interview (M.I.N.I.).

All BD subjects completed the rating scales and provided a 3-ml venous blood sample, which was immediately sent to the laboratory for the T lymphocyte subsets measurements. They also provided fecal samples to the State Key Laboratory for Diagnosis and Treatment of Infectious Diseases in the hospital. Sixteen patients completed the baseline test of the NIRS. As a first-line therapy for BD recommended by the Canadian Network for Mood and Anxiety Treatments and International Society for Bipolar Disorders 2018 guidelines (24), quetiapine was selected to treat BD patients in this study. All patients received a four-week treatment with quetiapine (from 50 mg/day gradually increase to 300 mg/day per person in 12 days) (25). During the treatment, no other psychotropic medications were used. Several nonpsychiatric drugs were used to ease side effects. Propranolol (10 mg–20 mg/day) was used to treat sinus tachycardia, and glycerine enema was used to treat constipation. No other medications were used. After 4 weeks, the rating scales were reassessed, and T lymphocyte subsets and fecal bacteria populations were retested. Seventeen patients completed the one-month follow up.

The HCs group also completed the rating scales for screening and provided fecal samples.

### Rating Scale Assessment for Mood Symptoms

In the BD group, depression severity was assessed using MADRS (21, 22). Mania severity was measured using YMRS (23). MADRS and YMRS were measured by two independent and experienced psychiatrists who were trained simultaneously in using the MADRS and YMRS before this study began. After training, a correlation coefficient greater than 0.8 was maintained for both the MADRS and the YMRS total score by repeated assessments. Both of the scales were evaluated twice, before and after treatment.

In the HCs group, MADRS, YMRS, and M.I.N.I. were used as screening tools. All the scores were below the lowest intervention thresholds that were aforementioned.

### Measurements of T Lymphocyte Subsets

Venous blood samples were collected from all patients (except one male patient who did not offer a blood sample). The proportions of T lymphocyte subsets (e.g., CD3^+^, CD4^+^, CD8^+^ T cells, and NK cells) were examined by a BD FACS CantoTM II flow cytometer (BD Bioscience, CA, United States; previously described in PMID: 8404602). The blood samples were stained with antibodies as follows: Pcy5-conjugated anti-CD3, FITC-conjugated anti-CD4, P-phycoerythrin-conjugated anti-CD8 (BD Biosciences, CA, United States), and PE-conjugated anti-CD16/CD56 (Beckman Coulter, CA, United States). These tests were all carried out in triplicate using the same batch of kits.

### Fecal Bacterial Population Determination

This study examined 10 common bacteria. *Faecalibacterium prausnitzii*, *Clostridium Cluster IV*, and *Eubacterium rectale* are butyrate producers (26–28). Sodium butyrate upregulates the level of brain-derived neurotrophic factor (BDNF) in the damaged brain (29). Levels of BDNF in the brain and serum are decreased in depressed patients (30). Thus, the fluctuation of gut *Faecalibacterium prausnitzii*, *Clostridium Cluster IV*, and *Eubacterium rectale* populations is responsible for the mood swing. *Enterococcus faecalis* is conditioned pathogen, and its metabolite, metalloprotease, is associated with the chronic intestinal inflammation (31). *Lactic acid bacteria* produces lactic acid. A peripheral intervention of lactate contributed to antidepressant-like effects with changes of epigenetics in hippocampus (32). Bifidobacteria is beneficial for the gut barrier, colonic immune system, and intestinal cell proliferation (33). *Enterobacter spp* have emerged as an important cause of nosocomial infections in clinic (34). Hence, we analyzed *Faecalibacterium prausnitzii*, *Clostridium Cluster IV*, *Eubacterium rectale*, *Enterococcus faecalis*, *Lactic acid bacteria*, and *Bifidobacteria* populations in feces of BD subjects. We also included *Bacteroides-Prevotella group*, *Clostridium Clusters I*, and *Atopobium Cluster* for population analysis. All of these bacteria were compared with those of HCs.

B/E, the gut *Bifidobacteria* to *Enterobacteriaceae* ratio, used to describe the microbial colonization resistance against pathogenic bacteria of the bowel (35–37). Its validity is widely accepted by researchers (36, 38, 39). The concept of colonization resistance (CR) was first put forward in 1971 by van der Waaij D et al. to describe the resistance that small amount of ingested Enterobacteriaceae was hard to colonize in animal intestinal tract (37). They describe CR as the endogenous anaerobic fraction of the intestinal microflora (37). Then, it was discussed again in 1992 by van der Waaij D (35). Based on this, researchers in State Key Laboratory for Diagnosis and Treatment of Infectious Diseases, First Affiliated Hospital, Zhejiang University School of Medicine, Zhongwen Wu et al. put forward a new index called B/E ratio to quantify microbial colonization resistance of the bowel (40). They also use it to evaluate the liver disease progression in a study reported in 2011 by Haifeng Lu, who is also a researcher in this laboratory (38). In 2004, B/E was used in an intestinal microecology study in irritable bowel syndrome patients (36). In 2018, B/E was used in a research studying how green tea polyphenols modulate gut microbiota (39). Therefore, in our study, the use of B/E is a new and rational attempt.

The fecal samples were divided into small samples in smaller tubes and were freshly preserved at -80°C within 30 min of defecation. DNA was extracted from the feces and precipitates with the Qiagen Stool Kit (Qiagen, Hilden, Germany), according to a modified protocol for cell lysis (41). DNA integrity was checked by agarose gel electrophoresis and UV-light imaging with ethidium bromide staining (38).

Though the sequencing of ribosomal targets using a single or limited set of primers is a more standard way to characterize the microbiota, qPCR and immunological techniques would be more suitable for clinical use. Therefore, we chose qPCR and immunological techniques to investigate 10 bacterial populations in the feces.

The process of qPCR was the same as the previous manipulations [see Lu et al. (38)]. The primers for qPCR were referenced from earlier studies (**Table 1**). All oligonucleotide primers were synthesized by TAKARA (Dalian, China).

**Table 1:** Primers used in the study.

Target group	Sequence (5′–3′)	Annealing temperature (°C)	References
**Faecalibacterium prausnitzii**	GATGGCCTCGCGTCCGATTAG CCGAAGACCTTCTTCCTCC	58	(41)
**Enterococcus faecalis**	AACCTACCCATCAGAGGG GACGTTCAGTTACTAACG	57	(41)
**Bacteroides–Prevotella group**	GAAGGTCCCCCACATTG CAATCGGAGTTCTTCGTG	56	(41)
**Lactic acid bacteria**	AGCAGTAGGGAATCTTCCA ATTYCACCGCTACACATG	58	(41)
**Bifidobacterium genus**	GGGTGGTAATGCCGGATG TAAGCCATGGACTTTCACACC	59	(41)
**Clostridium cluster IV**	GCACAAGCAGTGGAGT CTTCCTCCGTTTTGTCAA	50	(42)
**Clostridium clusters I**	TACCHRAGGAGGAAGCCAC GTTCTTCCTAATCTCTACGCAT	63	(43)
**Eubacterium rectale**	CGGTACCTGACTAAGAAGC AGTTT(C/T)ATTCTTGCGAACG	55	(44)
**Atopobium cluster**	GGGTTGAGAGACCGACC CGGRGCTTCTTCTGCAGG	55	(42)
**Enterobacteriaceae**	CATTGACGTTACCCGCAGAAGAAGC CTCTACGAGACTCAAGCTTGC	63	(41)

### NIRS Measurement

The activation task in this study was the verbal fluency task, which was similar to that used by Suto et al. in 2004 (45). Patients experienced a 30-s pretask baseline, 60-s activation task, and 70-s posttask baseline. For the pre and postbaseline periods, the individuals were instructed to consecutively repeat a train of numbers as follows: 1, 2, 3, 4, and 5. During the activation period, the individuals were instructed to generate as many Chinese terms as possible with specific initial Chinese characters. There were three sets of initial Chinese characters (“tian,” “da,” and “bai”), and every character changed every 20 s. The performance was determined by the number of correct terms generated by participants during the activation period.

**Figure 1:**
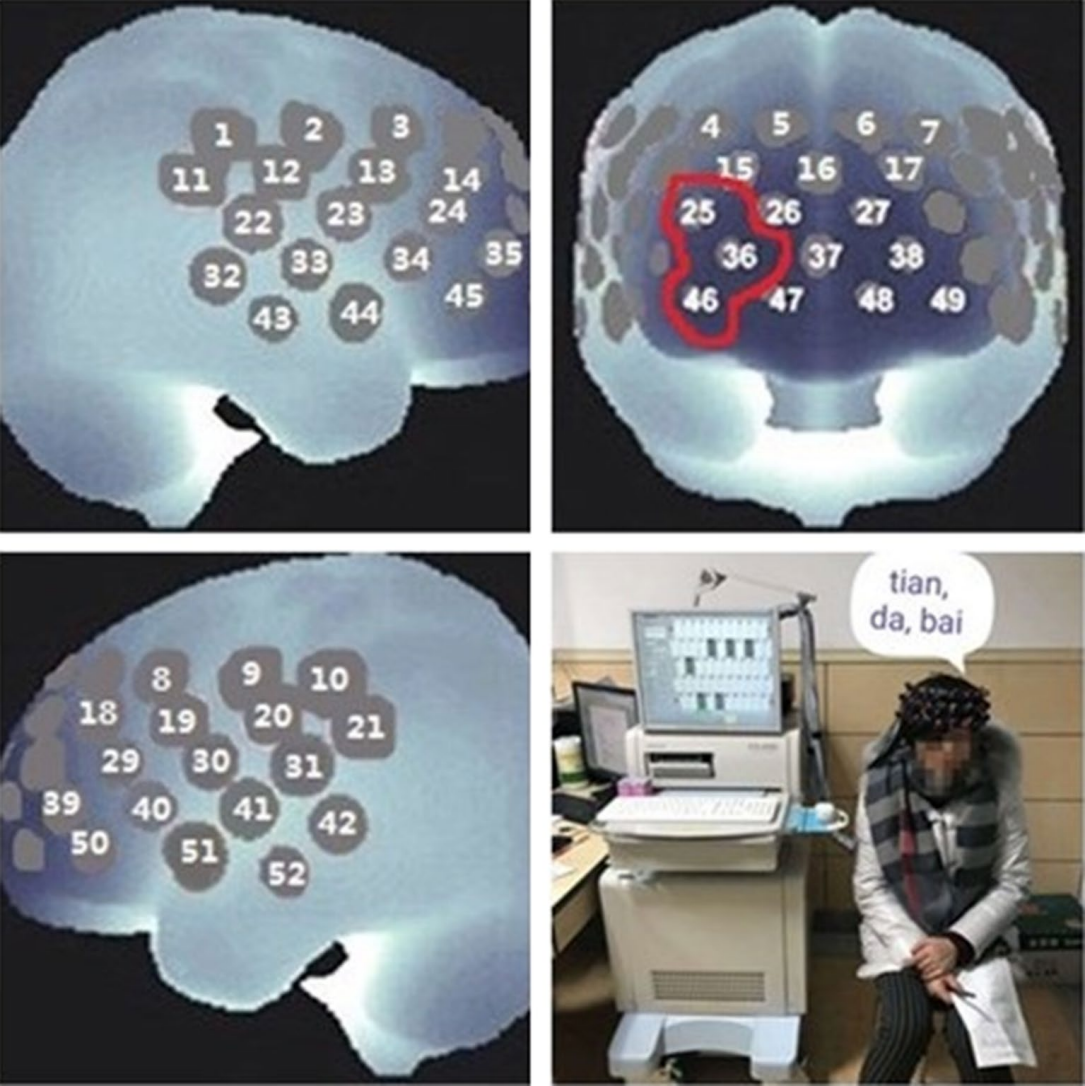
Near-infrared spectroscopy (NIRS) measurement. In this study, the [oxy-Hb] of the prefrontal cortex was examined by channel 25, channel 36, and channel 46, and the average was used as representative.

The NIRS system was a 52-channel one (ETG-4000; Hitachi Medical Co., Tokyo, Japan). A total of 33 probes consisted of 52 channels and covered the bilateral prefrontal and temporal regions (**Figure 1**). Among the 33 probes, there were 16 light emitters and 17 detectors with interoptrodes. Relative changes in oxy-Hb and deoxy-Hb were measured using two wavelengths (695 nm and 830 nm) of infrared light, based on the modified Beer-Lambert law (46–48). The temporal resolution was 0.1 sec. The distance between pairs of source-detector probes was set at 3.0 cm and the measured area involved by a pair of source-detector probes was defined as one “channel.” This machine is assumed to measure a “channel” at a depth of 2-3 cm from the scalp (i.e., the surface of the cerebral cortex).

The data was analyzed using the “integral mode” to examine the task-related activation. The pretask baseline was determined by the mean over a 10-s period instantly prior to the task period, and the posttask baseline was determined by the mean over the last 10 s of the posttask period. Linear fitting was performed between the two baseline datasets.

Both increased concentrations of oxygenated (oxy-Hb) and deoxygenated hemoglobin (deoxy-Hb) in micro-blood vessels reflected the cortical activation, and this was confirmed by comparing to other methods such as PET, SPECT, and functional MRI (45). The prefrontal function of bipolar patients is off-normal (49–52). Therefore, we took the changed absolute value of mean prefrontal oxy-Hb (deoxy-Hb) concentration, [oxy-Hb], to represent the prefrontal function. [oxy-Hb] was recorded during the 60-s task period. B/E, as a representative of the microbial colonization resistance, indicates the ability against pathogenic bacteria of the bowel. It is a character of gut microecological balance. As a growing body of evidences suggest the effects of gut microbiota on bipolar pathogenesis and refer to the brain-gut axis disturbance, we tried to connect gut microbiota to prefrontal function. We creatively postulated a parameter called brain-gut coefficient of balance (B-G_CB_) to assess the brain-gut correlations integrally. B-G_CB_ referred to the ratio of [oxy-Hb]/(B/E).

### Statistical Analysis

Statistical analysis was conducted using the SPSS 19.0 statistical software (IBM, IL, United States). All significance levels were two-tailed.

To compare the socio-demographic and clinical profiles, the χ^2^ test was applied to categorical variables, and analysis of variance (ANOVA) was used for the continuous variables.

To compare the gut bacterial populations between patients and HCs, ANOVA was used to test the gut microbiota differences. Once the ANOVA was significant, the effects of gender, BMI, age, education, and smoking were tested by adding these variables to the one-way analysis of covariance (ANCOVA). The same method was used for [oxy-Hb] comparison between groups.

For the baseline data of the BD group, correlation analysis among the T lymphocyte subsets, bacterial populations, and the MADRS score was performed. Before correlation analysis, each parameter was tested normality using Kolmogorov-Smirnov test with the correction of Lilliefors. Those abnormal distributed results were logarithmically transformed and then tested for normality again. The outliers were detected by box plots (95% confidence interval) and excluded. Pearson correlations were calculated for normally distributed data, and Spearman correlations were used for data that were not normally distributed. Bonferroni correction was used during the multiple comparisons.

The relationships between the log_10_ (B-G_CB_) and the severity of depression (MADRS score), the T lymphocyte subsets were explored using Pearson correlation analysis.

To analyze the therapeutic effect of four weeks’ treatment of quetiapine, the Mann-Whitney U test was used to compare the baseline and the posttreatment data. Every gut bacterial population was analyzed. Correlation analyses were performed between the declination rate of the MADRS score (the percentage of change from baseline to the end of the study) and the difference value of each bacterial population (or the difference values between the logarithmically transformed data) in order to determine the clinical predictive value of the gut flora to BD.

Gender differences in the gut microbiota in either of the groups were examined using the Mann-Whitney U test.

## Results

### Socio-Demographic and Clinical Characteristics

Comparing patients and HCs, no significant difference was found in age, gender, marital status, BMI, and handedness (*p* > 0.1), but the education level in the BD group was significantly different from that in HCs (*p* = 0.002). The other clinical information of the BD group was shown in **Table 2**. Socio-demographic information of patients who finished the follow up was shown in **Table 3**.

**Table 2:** The socio-demographic and clinical profiles of participants (mean ± SD).

	BD (n = 36, BD-I: n = 10, BD-II: n = 26)	HCs (n = 27)	χ*^2^* *or F*	*df*	p
**Age**	32.64 ± 10.643	28.89 ± 11.095	1.847	1	0.179^Δ^
**Gender, Male. %**	58.33	55.56	0.049	1	0.825^Δ^
**Marriage, Married. %**	61.11	44.44	1.725	1	0.189^Δ^
**Education (>12years) %**	44.44	81.48	10.364	1	0.002 *^Δ^
**Right-handed. %**	97.22	100	0.043	1	0.836^Δ^
**BMI**	22.16 ± 3.631	21.84 ± 3.092	0.135	1	0.714^Δ^
**BMI > 25 (overweight)**	25.00%	18.52%			
**Onset age**	26.06 ± 12.488	–			
**Duration of illness (year)**	7.40 ± 7.659	–			
**Number of episodes**	5.83 ± 4.583	–			
**N1**	17				
**N2**	17				
**N3**	19				
**N4**	16				

*p < 0.05 (two-tailed). ^Δ^Independent-samples t test. N1, number of patients who finished one-month follow up; N2, number of BD patients who was drug naive; N3, number of patients whose medications had been stopped for more than 3 months. N4, number of patients who finished the baseline NIRS measurement; BD, bipolar depression. BD¬I: type I bipolar depression. BD-II: type II bipolar depression; BMI, body mass index; HCs, healthy controls. SD, standard deviation.

**Table 3:** Socio-demographic information of patients who finished the follow up.

Items	Mean ± SD	Items	Percentage (%)
**Age**	33.59 ± 8.860	**Gender, Male.**	52.94
**BMI**	23.03 ± 3.757	**Marriage, Married.**	70.59
**Onset age**	26.24 ± 9.045	**BMI > 25 (overweight)**	29.41%
**Duration of illness (year)**	7.32 ± 6.915	**Education (>12years)**	47.06
**Number of episodes**	4.47 ± 2.294	**Right-handed.**	100
**Total number**	17		

BMI, body mass index; SD, standard deviation.

### Comparing Gut Microbiota Between the BD Patients and HCs

The counts of *Faecalibacterium prausnitzii*, *Bacteroides–Prevotella* group, *Atopobium Cluster*, *Enterobacter spp*, and *Clostridium Cluster IV* were significantly higher in the BD group compared with HCs (*p* = 0.030, *p* < 0.001, *p* < 0.001, *p* < 0.001, and *p* < 0.001, respectively, **Table 4**). Besides, the B/E ratio of the BD group was significantly lower than HCs (*p* = 0.001, **Table 4**).

**Table 4:** Bacterial populations in bipolar depression patients and healthy controls.

Bacteria	BD	HCs	*F*	*df*	*p*	p*
**Log_10_ Faecalibacterium prausnitzii**	7.01 ± 1.036	6.11 ± 0.892	2.545	6	0.001	0.030
**Log** **_10_** **Enterococcus faecalis**	4.17 ± 1.265	4.55 ± 1.377	.653	6	0.262	0.688
**Log** **_10_** **Bacteroides–Prevotella group**	8.70 ± 0.768	6.65 ± 1.139	14.372	6	<0.001	<0.001
**Log** **_10_** **Lactic acid bacteria**	4.12 ± 1.029	4.18 ± 1.239	.414	6	0.850	0.867
**Log** **_10_** **Bifidobacteria**	4.71 ± 1.187	4.95 ± 1.182	2.080	6	0.437	0.070
**Log** **_10_** **Clostridium Clusters I**	5.18 ± 1.048	4.89 ± 0.921	1.373	6	0.265	0.241
**Log** **_10_** **Eubacterium rectale**	6.07 ± 1.196	5.52 ± 0.804	1.374	6	0.045	0.241
**Log** **_10_** **Atopobium Cluster**	5.74 ± 0.758	4.96 ± 0.907	5.655	6	<0.001	< 0.001
**Log** **_10_** **Enterobacter spp**	6.07 ± 0.943	5.03 ± 0.746	6.137	6	<0.001	< 0.001
**Log** **_10_** **(B/E)**	–1.39 ± 1.157	–0.08 ± 1.005	4.381	6	<0.001	0.001
**Clostridium Cluster IV count**	8.54*10^7 ± 1.142*10^8	1.62*10^5 ± 1.249*10^5			<0.001△	

*Indicates adjusted p using analysis of covariance (ANCOVA), with adjustments made for age, gender, education, smoking, and body mass index (BMI). △Indicates Mann-Whitney U test for data that were not normally distributed. BD, bipolar depression; HCs, healthy controls; B/E, Bifidobacteria to Enterobacteriaceae ratio.

### The New Conception of the Brain-Gut Coefficient of Balance and Related Results

**Figure 2:**
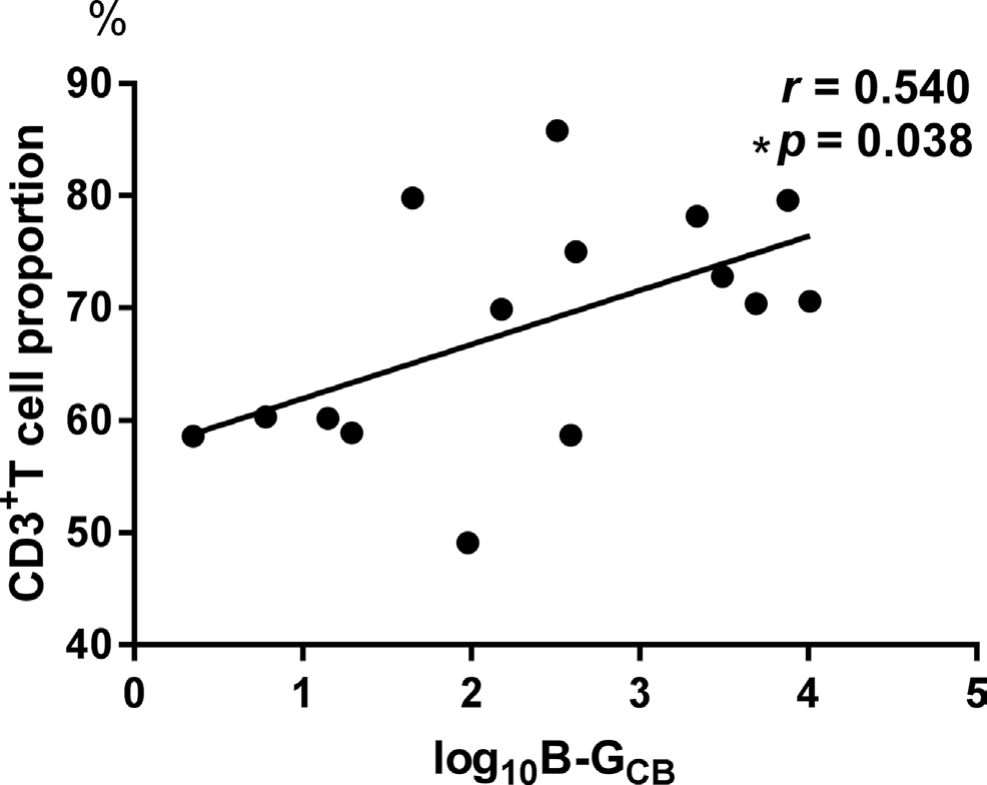
The log10 B-G_CB_ was positively correlated with the CD3^+^ T cell proportion (r = 0.540, p = 0.038).

The log_10_ B-G_CB_ was positively correlated with the CD3^+^ T cell proportion (*r* = 0.540, *p* = 0.038, **Figure 2**).

### Correlations Among Baseline Parameters Within the BD Group

We found that the CD3^+^ T cell proportion was positively correlated with log_10_ *Enterobacter spp* count (Bonferroni corrected p = 0.048, **Table 5**).

**Table 5:** Correlations among baseline parameters within the BD group.

	CD3^+^ T cell *p (r)*	CD4^+^ T cell	CD8^+^ T cell	NK cell	MADRS
**Log_10_ Faecalibacterium prausnitzii**	0.364(-0.158)	0.848 (0.034)	0.164 (-0.241)	0.551 (-0.104)	0.41 (0.141)
**Log_10_ Enterococcus faecalis**	0.518(0.113)	0.651 (-0.079)	0.127 (0.263)	0.408 (-0.144)	0.67 (-0.074)
**Log_10_ Bacteroides–Prevotella group**	0.488(-0.121)	0.538 (0.108)	0.091 (-0.29)	0.535 (-0.109)	0.343 (-0.163)
**Log_10_ Lactic acid bacteria**	0.665(0.076)	0.478 (0.124)	0.516 (-0.113)	0.77 (-0.051)	0.724 (0.061)
**Log_10_ Bifidobacteria**	0.743(-0.057)	0.57 (-0.1)	0.798 (0.045)	0.253 (0.198)	0.089 (0.288)
**Log_10_ Clostridium Clusters I**	0.126(-0.264)	0.536 (-0.108)	0.369 (-0.157)	0.975 (0.006)	0.754 (-0.054)
**Log_10_ Eubacterium rectale**	0.32(-0.173)	0.458 (0.13)	0.033 (-.362*)	0.576 (-0.098)	0.652 (-0.078)
**Log_10_ Atopobium Cluster**	0.619(0.087)	0.221 (0.212)	0.32 (-0.173)	0.077 (-0.303)	0.683 (0.071)
**Log_10_ Enterobacter spp**	0.004(.477**) 0.048△ *	0.364 (0.158)	0.027 (.374*) 0.324△	0.356 (-0.161)	0.493 (0.118)
**Log_10_ Clostridium Cluster IV count**	0.185(-0.229)	0.399(0.147)	0.045(-.341*) 0.54△	0.667(-0.075)	0.819(0.039)
**MADRS**	0.501(0.118)	0.849(-0.033)	0.658(0.077)	0.156(-0.245)	/

The unit of measure is p (r). We used Spearman correlations for LgClostridium Cluster IV count, and the others used Pearson correlations. p values < 0.05 are in bold. *p < 0.05. **p < 0.01. △ Corrected p, refers to Bonferroni corrected p. BD, bipolar depression; MADRS, the Montgomery-Asberg depression rating scale; NK cell, natural killer cell.

### Therapeutic Effect of Quetiapine

**Figure 3:**
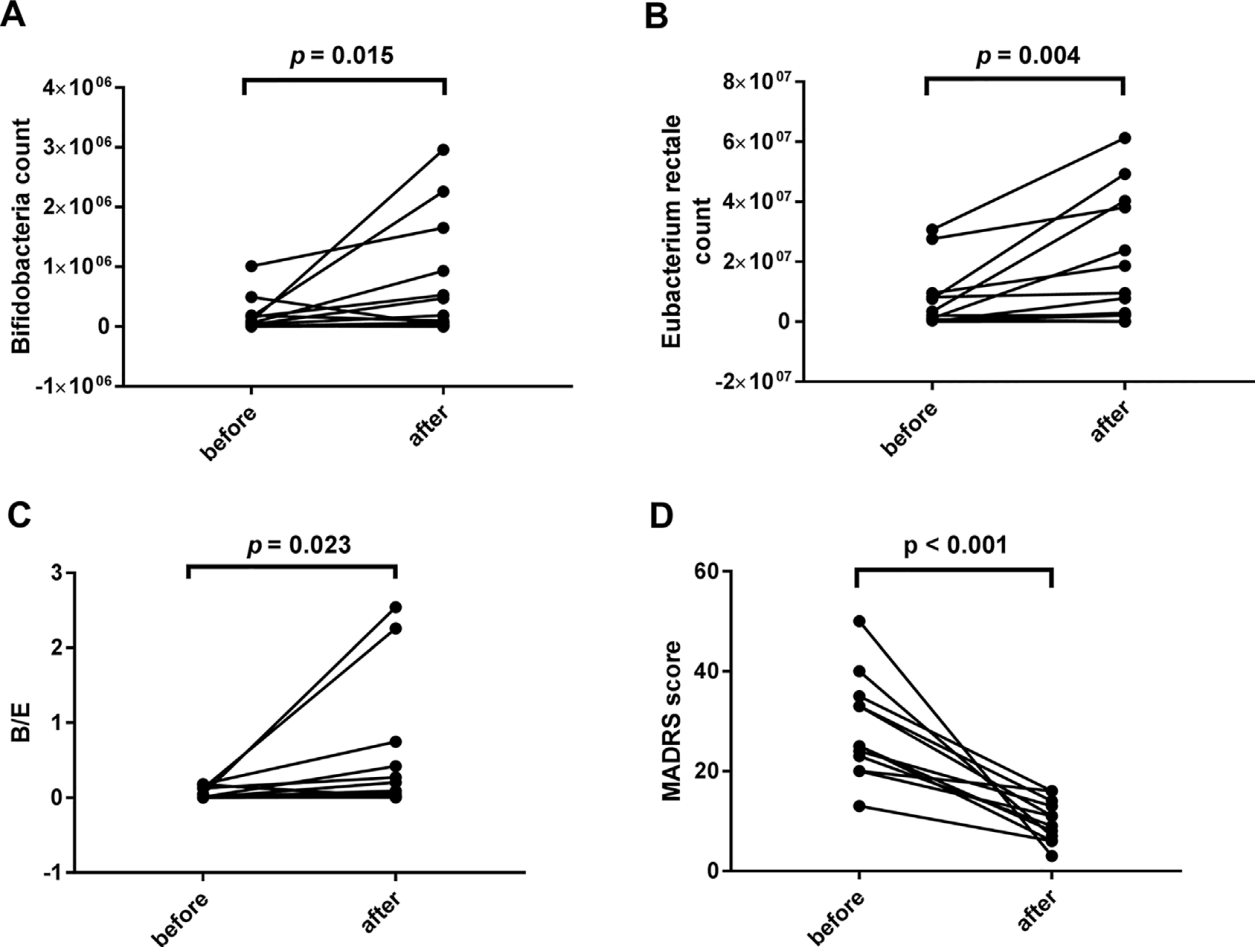
Comparisons between the baseline and the posttreatment data of bacterial populations and the Montgomery-Asberg depression rating scale (MADRS) score. Significant increases were found in the levels of *Bifidobacteria* count (1.33*10^5 ± 2.578*10^5 vs. 5.50*10^5 ± 8.968*10^5, *p* = 0.015), *Eubacterium rectale* count (5.58*10^6 ± 9.398*10^6 vs. 3.49*10^7 ± 7.955*10^7, *p* = 0.004), and B/E (0.0594 ± 0.07385 vs. 0.446 ± 0.8213, *p* = 0.023) **(A**–**C)**. The decrease of the MADRS score indicates a significant improvement in depressive symptoms (28.42 ± 10.166 vs. 10.00 ± 4.200, *p* < 0.001, **D**).

Posttreatment results were different from the baseline data (see **Figure 3**). However, no significant correlations were found between the declination rate of the MADRS scores and the bacteria quantity variations.

### Gender Difference in the Gut Microbiota

**Figure 4:**
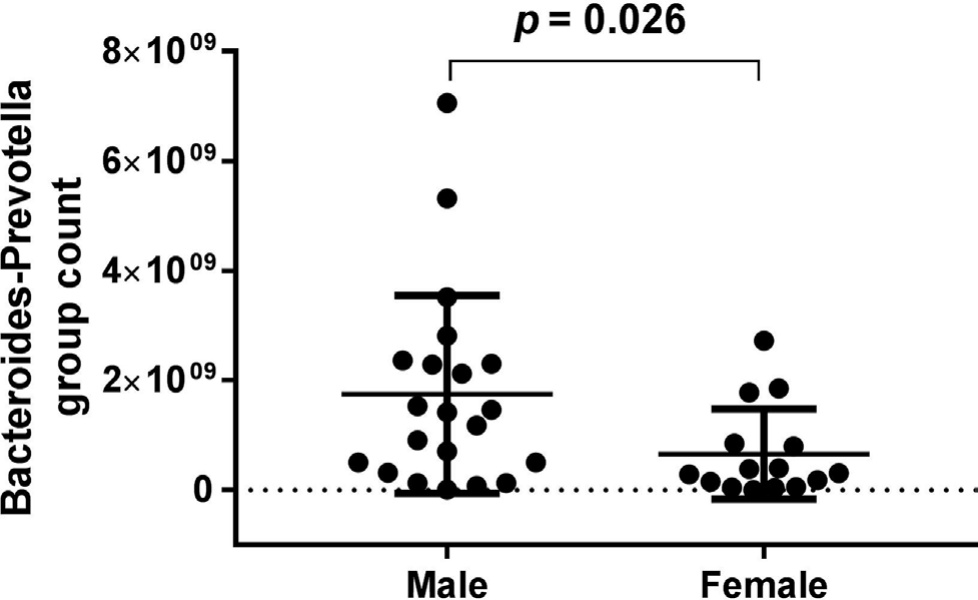
*Bacteroides-Prevotella* group count showed a gender difference in the bipolar depression (BD) group (male: 1.75*10^9 ± 1.801*10^9, female: 6.56*10^8 ± 8.213*10^8, *p* = 0.026) according to the baseline data.

*Bacteroides-Prevotella* group count showed a gender difference in the BD group (see **Figure 4**) according to the baseline data. However, in the HCs, none of the bacterial populations showed any gender difference (**Table 6**).

**Table 6:** Gender difference in the gut microbiota.

Bacteria	Group	Male (mean ± SD)	Female (mean ± SD)	*p*
**Faecalibacterium prausnitzii**	BD	3.29*10^7 ± 3.213*10^7	2.99*10^7 ± 3.380*10^7	0.619
	HCs	6.26*10^6 ± 1.134*10^7	9.47*10^6 ± 2.687*10^7	0.961
**Enterococcus faecalis**	BD	7.13*10^5 ± 1.976*10^6	4.14*10^4 ± 9.049*10^4	0.098
	HCs	1.32*10^6 ± 4.546*10^6	1.76*10^7 ± 6.059*10^7	0.329
**Bacteroides–Prevotella group**	BD	1.75*10^9 ± 1.801*10^9	6.56*10^8 ± 8.213*10^8	0.026*
	HCs	5.33*10^7 ± 9.105*10^7	2.35*10^7 ± 5.649*10^7	0.306
**Lactic acid bacteria**	BD	2.20*10^5 ± 5.211*10^5	5.31*10^4 ± 1.139*10^5	0.328
	HCs	2.02*10^6 ± 7.631*10^6	5.39*10^6 ± 1.864*10^7	0.922
**Bifidobacteria**	BD	5.43*10^5 ± 1.118*10^6	1.86*10^5 ± 2.596*10^5	0.531
	HCs	1.92*10^6 ± 3.356*10^6	1.51*10^5 ± 1.760*10^5	0.435
**Clostridium Cluster IV**	BD	1.11*10^8 ± 1.351*10^8	4.90*10^7 ± 6.410*10^7	0.061
	HCs	1.79*10^5 ± 1.354*10^5	1.42*10^5 ± 1.128*10^5	0.661
**Clostridium Clusters I**	BD	1.33*10^6 ± 2.465*10^6	5.87*10^5 ± 1.387*10^6	0.132
	HCs	9.59*10^5 ± 1.742*10^6	6.78*10^4 ± 7.702*10^4	0.157
**Eubacterium rectale**	BD	1.24*10^7 ± 2.782*10^7	6.93*10^6 ± 1.729*10^7	0.136
	HCs	2.41*10^6 ± 6.161*10^6	1.31*10^6 ± 2.904*10^6	0.770
**Atopobium Cluster**	BD	1.48*10^6 ± 1.686*10^6	1.61*10^6 ± 2.358*10^6	0.553
	HCs	8.41*10^5 ± 1.163*10^6	1.05*10^5 ± 1.389*10^5	0.088
**Enterobacter spp**	BD	5.54*10^6 ± 1.035*10^7	6.68*10^6 ± 1.179*10^7	0.748
	HCs	3.98*10^5 ± 5.201*10^5	2.23*10^5 ± 3.369*10^5	0.770
**B/E**	BD	0.563 ± .9231	0.096 ± 0.1467	0.344
	HCs	6.623 ± 10.8462	2.005 ± 3.0012	0.329

*p < 0.05 (two-tailed). B/E, Bifidobacteria to Enterobacteriaceae ratio. SD, standard deviation.

## Discussion

We found that among patients with BD: (1) counts of *Faecalibacterium prausnitzii*, *Bacteroides-Prevotella group*, *Atopobium Cluster*, *Enterobacter spp*, and *Clostridium Cluster IV* were significantly increased relative to HCs, whereas microbial colonization resistance was significantly decreased; (2) after treatment, populations of *Bifidobacteria* and *Eubacterium rectale* rebounded, and microbial colonization resistance recovered along with the decline of MADRS score; (3) B-G_CB_ was positively correlated with CD3^+^ T cell proportion; and (4) log_10_ (*Enterobacter spp* count) was positively correlated with CD3^+^ T cell proportion.

The gut microbiota composition in BD patients was different from that in HCs and was associated with illness severity and immune alterations. The expansion of *Bacteroides-Prevotella* group and Enterobacter spp indicates the dysbiosis of gut microbiota (53). The decrease of B/E indicated a weakened microbial colonization resistance. These results suggested that gut microecology was unbalanced in BD patients. The expansion of conditioned pathogen (e.g., *Enterobacter* spp) and pathogen (e.g., *Bacteroides-Prevotella* group) reduced the ability of intestine to resist exogenous pathogen. Former research finds a decreased cytotoxic cell level and an increased plasma IL-6 level (54) in bipolar patients. The immune activation in bipolar disorder patients is probably part of the results of the gut dysbiosis. This study found that Log_10_ (*Enterobacter spp* count) was positively correlated with CD3^+^ T cell proportion, indicating that *Enterobacter spp* expansion caused immune activation. A previous study shows a decrease in *Faecalibacterium* fractional representation in bipolar patients (16). However, our result found an elevated *Faecalibacterium prausnitzii* count. It seemed conflictive. The study design of Evans et al. was different from this study. Evans et al. recruited bipolar patients who were taking more than one psychotropic medication, whereas in this study, our patients had never received any psychotropic medcation or had stopped medication for more than 3 months. *Faecalibacterium prausnitzii* is a butyrate producer and has anti-inflammatory actions in colitis (55, 56). It helps to maintain the gut microbial balance. The discrepant results are probably associated with the different disease course and medication use. In this study, *Faecalibacterium prausnitzii* was supposed to be in a compensatory state without medication influence. *Clostridium Cluster IV* is another butyrate-producing bacteria (27) and belongs to beneficial bacteria. Therefore, in this study, beneficial bacteria populations expanded parallelly with pathogenic bacteria populations. This indicated that, in the early stage of the disease, gut bacteria changed follow a compensatory mechanism. They multiplied to protect gut resistance against the pathogen proliferation. The multiplied proportion was closely related to depressive severity.

The one-month quetiapine treatment was effective. The anaerobic *Bifidobacteria* and *Eubacterium rectale* proliferated during the one-month treatment. Microbial colonization resistance was determined by endogenous anaerobic bacteria fraction in the intestine (37). Anaerobic *Bifidobacteria* and *Eubacterium rectale* populations rebounded, suggesting the recovery of the microbial colonization resistance. This conclusion was supported by the posttreatment elevation of B/E.

B-G_CB_ is associated with immune disturbance when the brain-gut balance is broken. This study found that B-G_CB_ was positively associated with CD3^+^ T cell proportion. This suggested that BD subjects experienced an immune activation mediated by CD3^+^ T cell. As a T-cell co-receptor, CD3 helps to activate both the cytotoxic T-cells and T helper cells and is involved in signaling transduction between the inner and outside cytomembrane.

Similar to previous research findings, our study found that alterations in the composition of the gut microbiota may be associated with bipolar disorder and its severity (16). These BD specific bacteria can be used as screening biomarkers in clinic. *Bifidobacteria, Eubacterium rectale*, and B/E can be used as biomarkers to assess therapeutic effects. Our results found that CD3^+^ T cell mediated immune activation in BD patients. This points to future treatment with anti-inflammatory agents and probiotics in this population. Future studies are needed to confirm the utility of the B-G_CB_ in evaluating the course and treatment response in BD.

Several limitations to this study should be noted. First, the sample size is relatively small. This is not only a major limitation for the validity assessment of our findings but also for the subgroup analysis among different age groups. In this study, the wide age range of patients was downwards of 14 years old and upwards of 57 years old. It is clear that the gut microbiota of older adults differs from that of young people (57). Therefore, a larger sample size is required in future studies. Similarly, diet is an important factor that shapes the composition of gut microbiota (58). However, this report did not include any information regarding to diet. It is too difficult to analyze the impact of diet base on the small sample size and the single institution of patient source. Diet analysis is more feasible for multiregional cooperation studies. Second, 16S and metagenome sequencing are more suitable to explore the whole diversity of gut microbiota. The use of qPCR is another major limitation in this study. This should be improved in the following studies. Third, the patient group did not receive placebo treatment as a control. *Eubacterium rectale* count, *Bifidobacteria* count, and B/E (*Bifidobacteria* to *Enterobacteriaceae* ratio, microbial colonization resistance) elevated. It is hard to distinguish whether the elevation is due to the quetiapine treatment or the natural disease course. Fourth, we did not collect blood and fecal samples of HCs at four-week treatment. This study did not compare fecal microbiota of BD group and HCs at four-week treatment. Therefore, this research could not explain whether the four-week treatment makes the gut microbiota of patients approaching that of HCs. Fifth, BMI of BD group was lacking at four-week treatment. Therefore, this study failed to help patients’ BMI management during quetiapine treatment. Exogenous bacteria intake may benefit the patients’ BMI. Further study is needed to find the exact bacteria associated with drug-induced obesity. Atypical antipsychotics treatment causes weight gain and decreases the richness of gut microbiota (19, 59, 60). This is more remarkable in females and is closely related to drug dose (19, 59). High dose impacts more bacteria species. Olanzapine treatment using 2 mg/kg increases *Firmicutes* level, decreases *Actinobacteria* level in female rats, and reduces *Proteobacteria* level in both sexes (59). Olanzapine treatment using 4 mg/kg reduces both *Actinobacteria* and *Proteobacteria* levels in female rats, increases *Firmicutes* level, and decreases *Bacteriodetes* level in both sexes (59). In a human study, atypical antipsychotics reduces species diversity of gut bacteria in female patients. The involved genera includes *Lachnospiraceae*, *Akkermansia*, and *Sutterella* (19). Therefore, this study could not clarify the reason why *Eubacterium rectale* count, *Bifidobacteria* count, and B/E elevated. It is probably because of the recovery of the disease or just the influence of quetiapine. The fecal re-examination is needed for the follow-up BD patients with placebo treatment or who reject treatments.

In summary, this study found that the composition of the gut microbiota in BD subjects was altered. The alteration of gut microbiota was associated with immune activation and linked to brain function. Quetiapine treatment was effective for the remission of depression and influenced the gut microbiota in patients. Further studies are required to confirm our findings and achieve more clinically useful results.

## Data Availability Statement

All datasets generated for this study are included in the article/supplementary Material.

## Ethics Statement

The studies involving human participants were reviewed and approved by Ethics Committee of the First Affiliated Hospital, Zhejiang University School of Medicine. Written informed consent to participate in this study was provided by the participants’ legal guardian/next of kin.

## Author Contributions

SH designed the trail and directed the entire study. YX, ZL directed the study togethor with SH. QL performed the study, analyzed the results, and wrote the manuscript. HL and HZ tested the gut microbiome. CN modified the manuscript. ZW helped with the manuscript revision and the data analysis. All authors made substantive contribution to this paper and approved the final manuscript.

## Funding

This study was supported by the grants of the National Natural Science Foundation of China (81671357, 81971271 to SH), the National Natural Science Foundation of China (81771474 to YX), the National Key Research and Development Program of China (No. 2016YFC1307104 to SH), the general project supported by Hangzhou Health Commission (2015A32 to KD) and Science and Technology Department of Zhejiang Province (2017C37037 to NW).

## Conflict of Interest

The authors declare that the research was conducted in the absence of any commercial or financial relationships that could be construed as a potential conflict of interest.

## Abbreviations

ANCOVA, analysis of covariance; ANOVA, analysis of variance; BD, bipolar depression; BDNF, brain-derived neurotrophic factor; B/E, Bifidobacteria to Enterobacteriaceae ratio; B-GCB, brain-gut coefficient of balance; CNS, central nervous system; deoxy-Hb, concentration of deoxygenated hemoglobin; HCs, healthy controls; MADRS, Montgomery-Asberg depression rating scale; M.I.N.I., the mini-international neuropsychiatric interview; NIRS, near-infrared spectroscopy; oxy-Hb, concentration of oxygenated hemoglobin; qPCR, quantitative polymerase chain reaction; SCFAs, short-chain fatty acids; SCID, structured clinical interview for DSM-IV-TR disorders; YMRS, young mania rating scale; 5-HT, 5-hydroxytryptamine.
